# cPAS-based sequencing on the BGISEQ-500 to explore small non-coding RNAs

**DOI:** 10.1186/s13148-016-0287-1

**Published:** 2016-11-21

**Authors:** Tobias Fehlmann, Stefanie Reinheimer, Chunyu Geng, Xiaoshan Su, Snezana Drmanac, Andrei Alexeev, Chunyan Zhang, Christina Backes, Nicole Ludwig, Martin Hart, Dan An, Zhenzhen Zhu, Chongjun Xu, Ao Chen, Ming Ni, Jian Liu, Yuxiang Li, Matthew Poulter, Yongping Li, Cord Stähler, Radoje Drmanac, Xun Xu, Eckart Meese, Andreas Keller

**Affiliations:** 1 Clinical Bioinformatics, Saarland University, 66125 Saarbrücken, Germany; 2 BGI-Shenzhen, Shenzhen, China; 3 Department of Human Genetics, Saarland University, Saarbrücken, Germany; 4 Complete Genomics (a BGI company), Mountain View, CA USA

**Keywords:** Next-generation sequencing, miRNA, Biomarker discovery, BGISEQ

## Abstract

**Background:**

We present the first sequencing data using the combinatorial probe-anchor synthesis (cPAS)-based *BGISEQ-500* sequencer. Applying cPAS, we investigated the repertoire of human small non-coding RNAs and compared it to other techniques.

**Results:**

Starting with repeated measurements of different specimens including solid tissues (brain and heart) and blood, we generated a median of 30.1 million reads per sample. 24.1 million mapped to the human genome and 23.3 million to the *miRBase*. Among six technical replicates of brain samples, we observed a median correlation of 0.98. Comparing BGISEQ-500 to HiSeq, we calculated a correlation of 0.75. The comparability to microarrays was similar for both BGISEQ-500 and HiSeq with the first one showing a correlation of 0.58 and the latter one correlation of 0.6. As for a potential bias in the detected expression distribution in blood cells, 98.6% of HiSeq reads versus 93.1% of BGISEQ-500 reads match to the 10 miRNAs with highest read count. After using miRDeep2 and employing stringent selection criteria for predicting new miRNAs, we detected 74 high-likely candidates in the cPAS sequencing reads prevalent in solid tissues and 36 candidates prevalent in blood.

**Conclusions:**

While there is apparently no ideal platform for all challenges of miRNome analyses, cPAS shows high technical reproducibility and supplements the hitherto available platforms.

**Electronic supplementary material:**

The online version of this article (doi:10.1186/s13148-016-0287-1) contains supplementary material, which is available to authorized users.

## Background

Currently, high-throughput analytical techniques are massively applied to further the understanding of the non-coding transcriptome [1]. Still, the full complexity of non-coding RNAs is only partially understood. One class of well-studied non-coding RNAs comprises small oligonucleotides, so-called miRNAs [2, 3].

Among the techniques most commonly used for miRNA profiling are microarrays, RT-qPCR, and next-generation sequencing (NGS), also referred to as high-throughput sequencing (HTS). An excellent review on the different platforms and a cross-platform comparison has been recently published [4]. A detailed examination of technologies, however, frequently reveals a bias. One reason for the respective bias is the ligation step, as, e.g., reported by Hafner and co-workers [5]. For example, the quantification of miRNAs differs between NGS and microarrays as it is dependent on base composition [6]. Especially, the guanine and uracil content of a miRNA seems to influence the abundance depending on the platform used. A substantial strength of NGS is the ability to support the completion of the non-coding transcriptome. Unlike microarrays and RT-qPCR, NGS allows the discovery of novel miRNA candidates. To this end, different algorithms have been implemented, with *miRDeep* being one of the most popular ones [7]. A substantial part of small RNA sequencing data has been obtained using HiSeq and MiSeq platforms (Illumina) based on stepwise sequencing by polymerase on DNA microarrays prepared by bridge PCR [8], as well as the IonTorrent systems from Thermo Fisher Scientific using a different type of polymerase-based stepwise sequencing on micro-bead arrays generated by emulsion PCR, the first method proposed for making microarrays for massively parallel sequencing [9]. Another approach is the ligase-based stepwise sequencing also using micro-bead arrays, applied for example by ThermoFisher Scientific’s SOLiD sequencing platform, and which has also been used to analyze and present novel miRNAs [10].

In the current study, we applied the new combinatorial probe-anchor synthesis (cPAS)-based BGISEQ-500 sequencing platform that combines DNA nanoball (DNB) nanoarrays [11] with stepwise sequencing using polymerase. An important advantage of this technique compared to the previously mentioned sequencing systems is in that no PCR is applied in preparing sequencing arrays. Applying cPAS, we investigated the human non-coding transcriptome. We first evaluated the reproducibility of sequencing on standardized brain and heart samples, then compared the performance to Agilent’s microarray technique and finally evaluated blood samples. Using the web-based miRNA analysis pipeline *miRmaster* and the tool *novoMiRank* [12], we finally predicted 135 new high-likely miRNA candidates specific for tissue and 35 new miRNA candidates specific for blood samples.

## Methods

### Samples

In this study, we examined the performance of three sample types using three techniques for high-throughput miRNA measurements (Illumina’s HiSeq sequencer, Agilent’s miRBase microarrays, and BGI’s BGISEQ-500 sequencing system, see details below). The three specimens were standardized HBRR sample ordered from Ambion (catalog number AM6051) and UHRR sample ordered from Agilent (catalog number 740000). UHRR and HBRR samples were measured in two and six replicates, respectively. As third sample type, we used *PAXGene* blood tubes. Here, two healthy volunteers’ blood samples were collected and miRNAs were extracted using PAXgene Blood RNA Kit (Qiagen) according to manufacturer’s protocol. The study has been approved by the local ethics committee.

### Next-generation sequencing using BGISEQ-500

We prepared the libraries starting with 1 μg total RNA for each sample. Firstly, we isolated the microRNAs (miRNA) by 15% urea-PAGE gel electrophoresis and cut the gel from 18 to 30 nt, which corresponds to mature miRNAs and other regulatory small RNA molecules. After gel purification, we ligated the adenylated 3′ adapter to the miRNA fragment. Secondly, we used the RT primer with barcode to anneal the 3′ adenylated adapter in order to combine the redundant unligated 3′ adenylated adapter. Then, we ligated the 5′ adapter and did reverse transcript (RT) reaction. After cDNA first strand synthesis, we amplified the product by 15 cycles. We then carried out the second size selection operation and selected 103–115 bp fragments from the gel. This step was conducted in order to purify the PCR product and remove any nonspecific products. After gel purification, we quantified the PCR yield by Qubit (Invitrogen, Cat No. Q33216) and pooled samples together to make a single strand DNA circle (ssDNA circle), which gave the final miRNA library.

DNA nanoballs (DNBs) were generated with the ssDNA circle by rolling circle replication (RCR) to enlarge the fluorescent signals at the sequencing process as previously described [11]. The DNBs were loaded into the patterned nanoarrays and single-end read of 50 bp were read through on the BGISEQ-500 platform for the following data analysis study. For this step, the BGISEQ-500 platform combines the DNA nanoball-based nanoarrays [11] and stepwise sequencing using polymerase, as previously published [13–15]. The new modified sequencing approach provides several advantages, including among others high throughput and quality of patterned DNB nanoarrays prepared by linear DNA amplification (RCR) instead of random arrays by exponential amplification (PCR) as, e.g., used by Illumina’s HiSeq and longer reads of polymerase-based cycle sequencing compared to the previously described combinatorial probe-anchor ligation (cPAL) chemistry on DNB nanorrays [11]. The usage of linear DNA amplification instead of exponential DNA amplification to make sequencing arrays results in lower error accumulation and sequencing bias.

### Next-generation sequencing using HiSeq

Samples have been sequenced using Illumina HiSeq sequencing according to manufacturer’s instructions and as previously described [16, 17].

### Agilent microarray measurements

For detection of known miRNAs, we used the SurePrint G3 8×60k miRNA microarray (miRBase version 21, Agilent Technologies) containing probes for all miRNAs from miRBase version 21 in conjunction with the miRNA Complete Labeling and Hyb Kit (Cat. No. 5190-0456) according to the manufacturer’s recommendations. In brief, 100 ng total RNA including miRNAs was dephosphorylated with calf intestine phosphatase. After denaturation, Cy3-pCp was ligated to all RNA fragments. Labeled RNA was then hybridized to an individual 8×60k miRNA microarray. After washing, array slides were scanned using the Agilent Microarray Scanner G2565BA with 3-μm resolution in double-pass mode. Signals were retrieved using Agilent AGW Feature Extraction software (version 10.10.11).

### Data availability

The new sequencing data using BGISEQ-500 data are available in the Additional file of this manuscript (Additional file 1: Table S3).

### Bioinformatics analysis

The raw reads were collapsed and used as input for the web-based tool miRMaster, allowing for integrated analysis of NGS miRNA data. On the server side, mapping to the human genome was carried out using *Bowtie* [18] (one mismatch allowed). miRNAs were quantified similar to the popular *miRDeep2* [19] algorithm. The prediction of novel miRNAs was performed using an extended feature set built up on novoMiRank [12]. For classification, an *AdaBoost* model using decision trees was applied. Novel miRNAs were cross-checked against other RNA resources, including the *miRBase* [20], *NONCODE2016* [21], and *Ensembl* non-coding RNAs. The assessment of the quality of new miRNAs was carried out using the novoMiRank algorithm. A downstream analysis of results including cluster analysis was performed using R. For target prediction, we applied TargetScan 7.1 (http://www.targetscan.org/vert_71/) and predicted for all new miRNAs the targets. With the predictions, we extracted the context++ scores and used them for prioritizing the targets, miRNA-target interactions with context++ scores below 1 were considered as high-likelihood targets. Target networks were constructed using an offline version of MiRTargetLink [22] and visualized in Cytoscape. miRNA target pathway analysis has been carried out using GeneTrai2 [23]. For the GeneTrail2 analysis, all available categories were analyzed, the minimal category size was set to 4 and all *p* values were adjusted using Benjamini-Hochberg adjustment.

## Results

### Raw data analysis

We sequenced six brain, two heart, and two blood samples using the BGISEQ-500 system. The resulting reads were mapped to the human genome allowing one mismatch per read. The 10 samples had a median of 30.1 million reads. Of these, 24.1 million reads mapped to the human genome and 23.3 million reads to miRNAs annotated in the human miRBase version 21. The remaining 0.7 million reads per sample contain potentially new miRNAs.

### Technical reproducibility of the BGISEQ-500 and comparison to microarrays

**Fig. 1:**
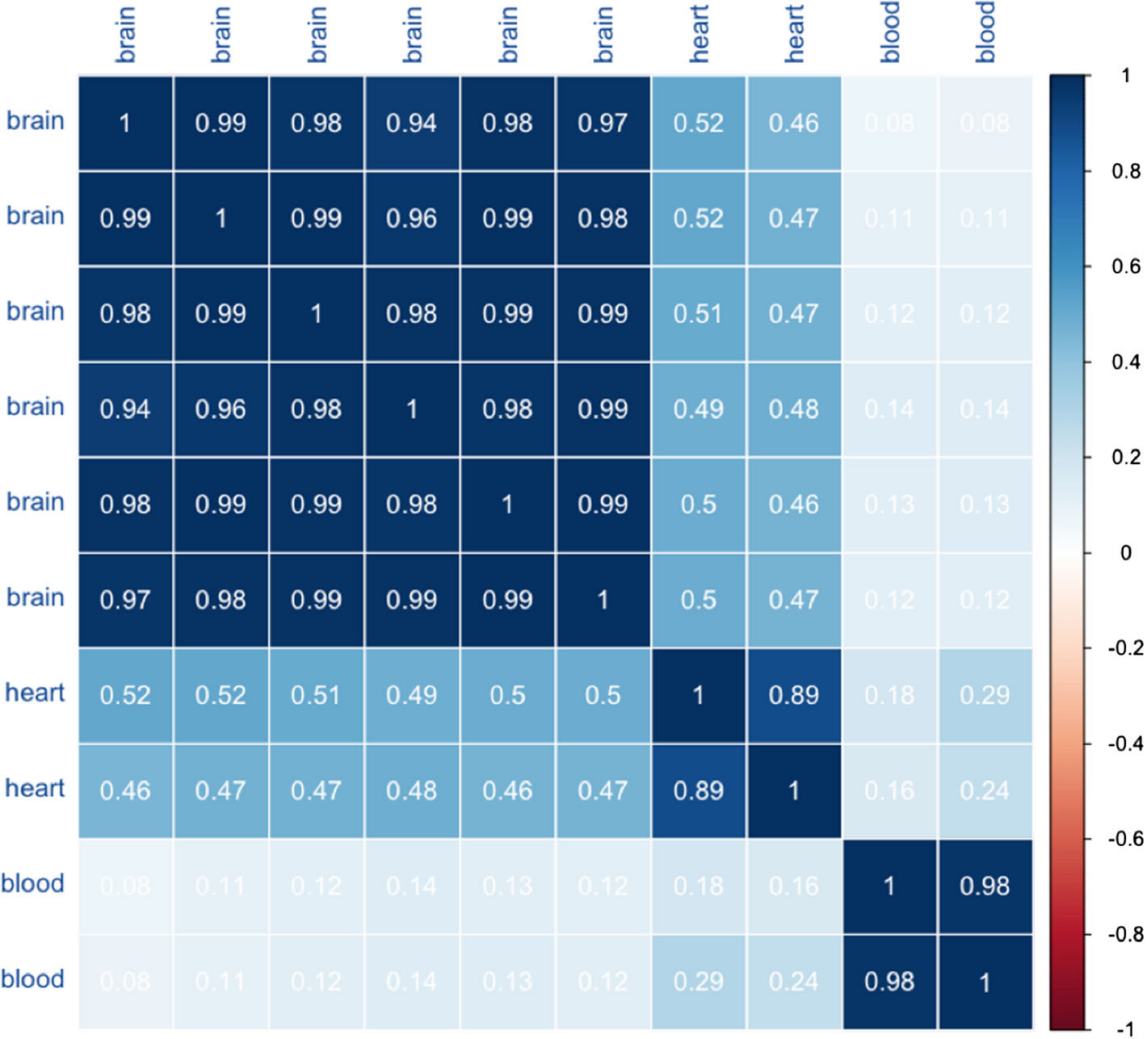
Correlation matrix of the brain (six technical replicates), heart (two technical replicates), and blood (two biological replicates) sequenced by the BGISEQ-500 system

To assess the technical reproducibility of the sequencing platform, we evaluated the six technical replicates of the human brain sample (see correlation matrix in Fig. 1). The median correlation between the six replicates was 0.98, and the 25 and 75% quantile were 0.98 and 0.99, respectively. These data suggest an overall high correlation for technical replicates on the BGISEQ-500 platform.

**Fig. 2:**
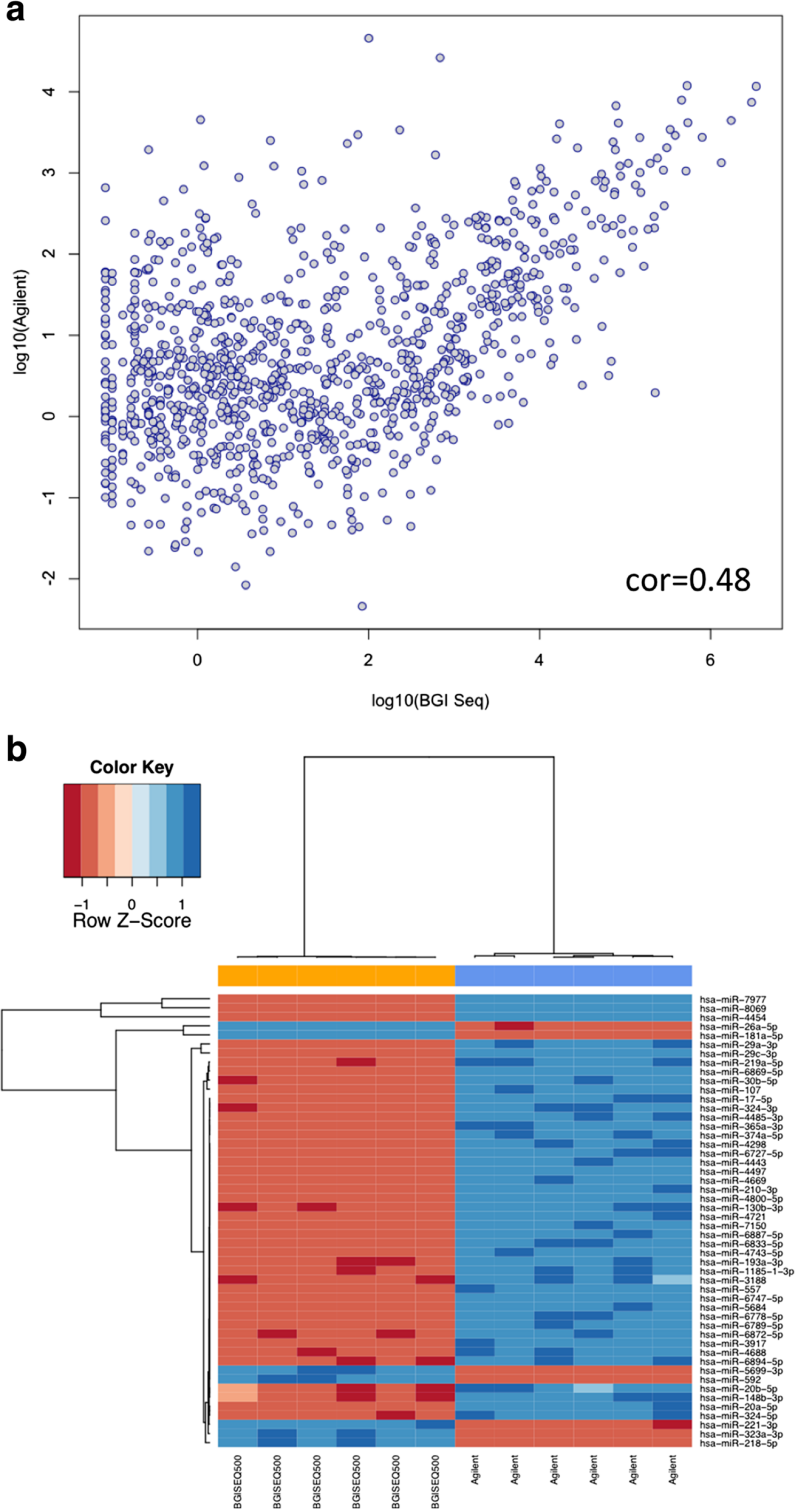
**a** Log average expression of common miRNAs for the brain RNA on BGISEQ-500 and on Agilent microarrays (six technical replicates each). **b** Heat map with dendrogram for the 50 most differently detected miRNAs in the brain RNA between Agilent and BGISEQ-500 (six technical replicates each)

Comparing the BGISEQ-500 data to the measurements of the brain sample with microarrays (miRBase version 21) that have also been carried out as six technical replicates (median correlation of the microarrays was 0.999), we observed a log correlation of 0.48. A direct comparison is presented in the scatter plot in Fig. 2a. This plot highlights many miRNAs that can be measured at a comparable level on both platforms. However, a subset of the small non-coding RNAs is shifted towards higher expression on the array platform. The same behavior can be observed in the cluster heat map in Fig. 2b. This heat map graphically represents the 50 miRNAs with most different detection between both techniques. To compare rather the ranks of miRNAs instead of the absolute read counts, the replicated brain samples on both platforms were jointly quantile normalized. Three miRNAs, in particular, showed highly significant deviations (multiple testing adjusted *p* values below 10^−20^). Hsa-miR-8069 was almost not detected in the BGISEQ-500 but had 0.9 million normalized intensity counts on the array platform, hsa-miR-4454 had 51.6 normalized reads on the BGISEQ-500 versus 1.9 million normalized counts on the microarrays, and hsa-miR-7977 had 343.2 normalized reads on the BGISEQ-500 versus 1.3 million normalized counts on the microarrays. This means that the three miRNAs were orders of magnitudes more abundant on microarrays as compared to the sequencing system. The secondary structures of the three precursors are presented in Additional file 2: Figure S1. These results match well to previously published platform comparisons between NGS and microarrays [6]. Here, several miRNAs such as hsa-miR-941 (not detected in any array experiment, not detected in RT-qPCR, average read count of ~1000 reads using Illumina HiSeq sequencing) had expression levels differing several orders of magnitude between the miRBase microarrays and using HiSeq sequencing.

The full list of miRNAs with raw and adjusted *p* values in *t* test and Wilcoxon-Mann-Whitney test comparing BGISEQ-500 and microarrays is presented in Additional file 3: Table S1. Overall, the results are well in-line with those obtained between HiSeq NGS and the same microarray platform [6]. Reasons that explain differences between arrays and NGS include different sensitivity levels of the platforms, cross-hybridization of miRNAs with similar sequences on the microarrays or bias in library preparation. Further, effects of the normalization can lead to variations in miRNA quantification.

### Biological replicates of blood samples and comparison to other platforms

**Fig. 3:**
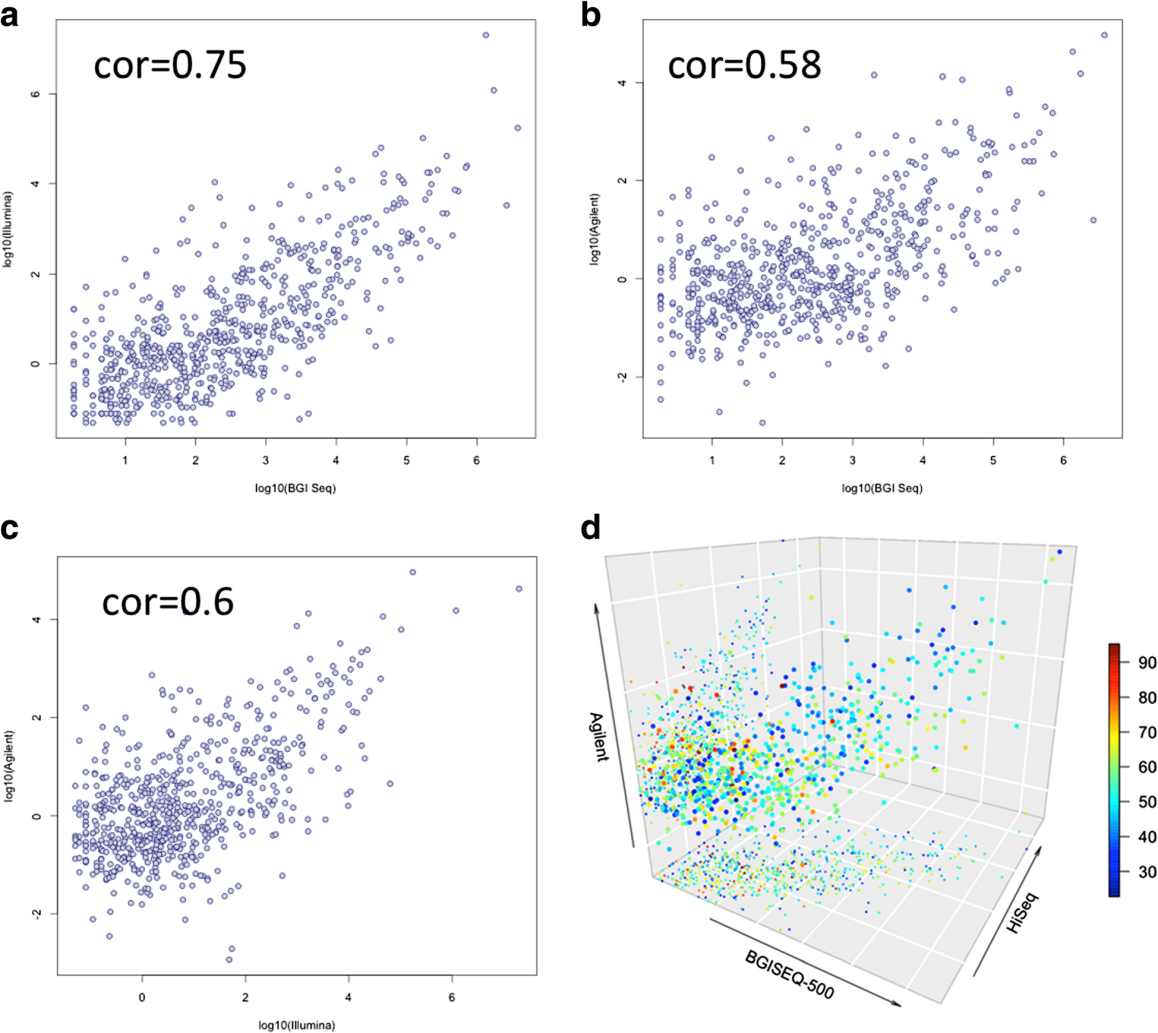
**a**-**c** Pairwise scatter plots for comparing expression of miRNAs in blood cells on microarrays, HiSeq, and BGISEQ-500. Please note that for HighSeq and Agilent technical replicates were measured, for BGISEQ-500 biological replicates. **d** 3D scatter-plot colored by the GC content of miRNAs

One of the most promising applications in small RNA analysis is biomarker profiling in body fluids. We previously analyzed over 2000 blood samples on Agilent microarrays [17, 24, 25] and about 1000 samples using HiSeq sequencing [26, 27] and compared both platforms [6]. We correlated two newly sequenced blood samples using the BGISEQ-500 system to the data generated by HiSeq and Agilent microarrays. When interpreting the results, it is important to keep in mind that the microarrays and HiSeq data are from the same samples [6] while the newly sequenced blood drawings are from other individuals and thus biological but no technical replicates. To minimize a potential bias between the platforms with respect to different miRNA sets, we first reduced the marker set to the 2525 human miRNAs that were profiled on all platforms and next to the subset of 658 miRNAs that were discovered in all three platforms. For each, platform data were normalized using quantile normalization. Due to the wide dynamic range of miRNAs in blood samples, which is approximately 10^7^, we present the three pairwise comparisons (BGISEQ-500 to microarrays, BGISEQ-500 to HiSeq, and HiSeq to microarrays) on a log scale. The scatter plots are presented in Fig. 3. The highest correlation was observed for BGISEQ-500 to Illumina (0.75, Fig. 3a). Even the correlation between microarrays and HiSeq was below this value (0.6, Fig. 3c). Especially since technical replicates have been measured for these platforms, the increased correlation of sequencing platforms is remarkable. The comparison of BGISEQ-500 and microarrays revealed correlation values in the same range as for the brain samples (0.58, Fig. 3b). The 3D scatter plot in Fig. 3d compares the expression of the three platforms directly to each other. The coloring of the miRNAs has been carried out with respect to the GC content.

### Expression distribution of miRNAs

**Fig. 4:**
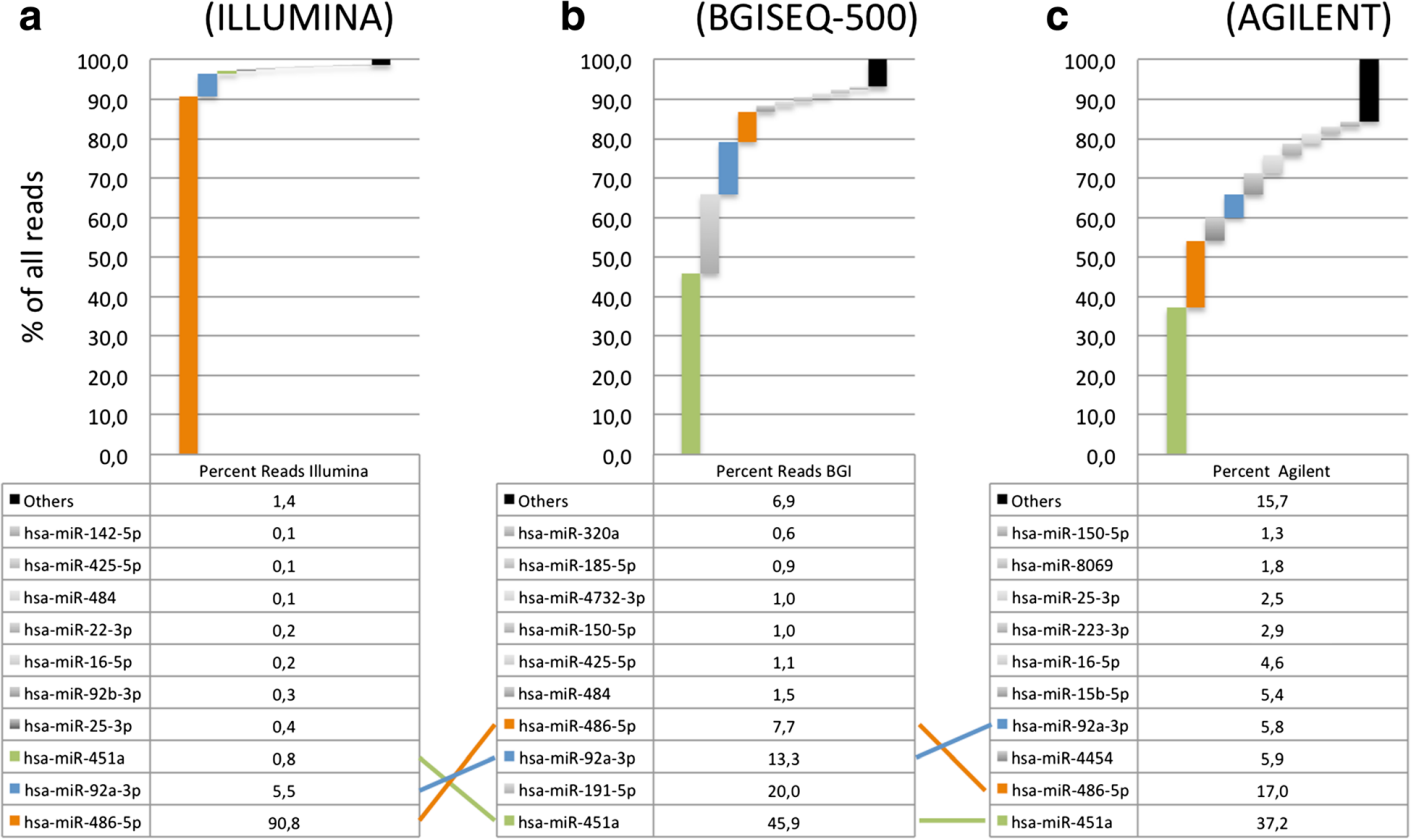
Expression distribution of the 10 miRNAs with the highest detection in the blood RNA on the HiSeq system (**a**), BGISEQ-500 (**b**), and microarray system (**c**). Note that for the Agilent microarray system, the sum of all expression intensities was assumed to be 100%

As mentioned, miRNA expression is highly variable and can scatter across many orders of magnitude. We thus compared the distribution of the sequencing reads in blood samples on the HiSeq to the BGISEQ-500. Blood samples, including blood cells (especially red blood cells) are known to be enriched for few miRNAs that are highly expressed. The diagram in Fig. 4 (panel A) highlights that 90.8% of all blood sequencing reads from the HiSeq match to one single miRNA: hsa-miR-486-5p. The second most abundant miRNA miR-92a-3p takes further 5.5%, and already the third most abundant marker miR-451a has below 1% of all reads. In sum, 98.6% of all reads match to the top 10 miRNAs. For the BGISEQ-500 (panel B), 45.9% of reads match to miR-451a, further 20% map to miR-191-5p and 13.3% map to miR-92a-3p. The most abundant miRNA in HiSeq, miR-486-5p, is detected in 7.7% of all reads. 93.1% of all sequenced reads match to the top 10 miRNAs.

Comparison of the distribution and abundance of miRNAs on the microarray platform is difficult since microarrays show a saturation effect. This means that for two miRNAs expressed in a range above the saturation, no difference can be observed. We nonetheless performed the same analysis as presented above, assuming that the sum of all expression counts equals to 100%. In this analysis, miR-451a which is found in 0.8% of HiSeq reads and 45.9% of BGISEQ-500 reads is the highest expressed in microarrays (37.2% of all expression counts), followed by 17% of miR-486-5p.

### Prediction of novel miRNAs

**Fig. 5:**
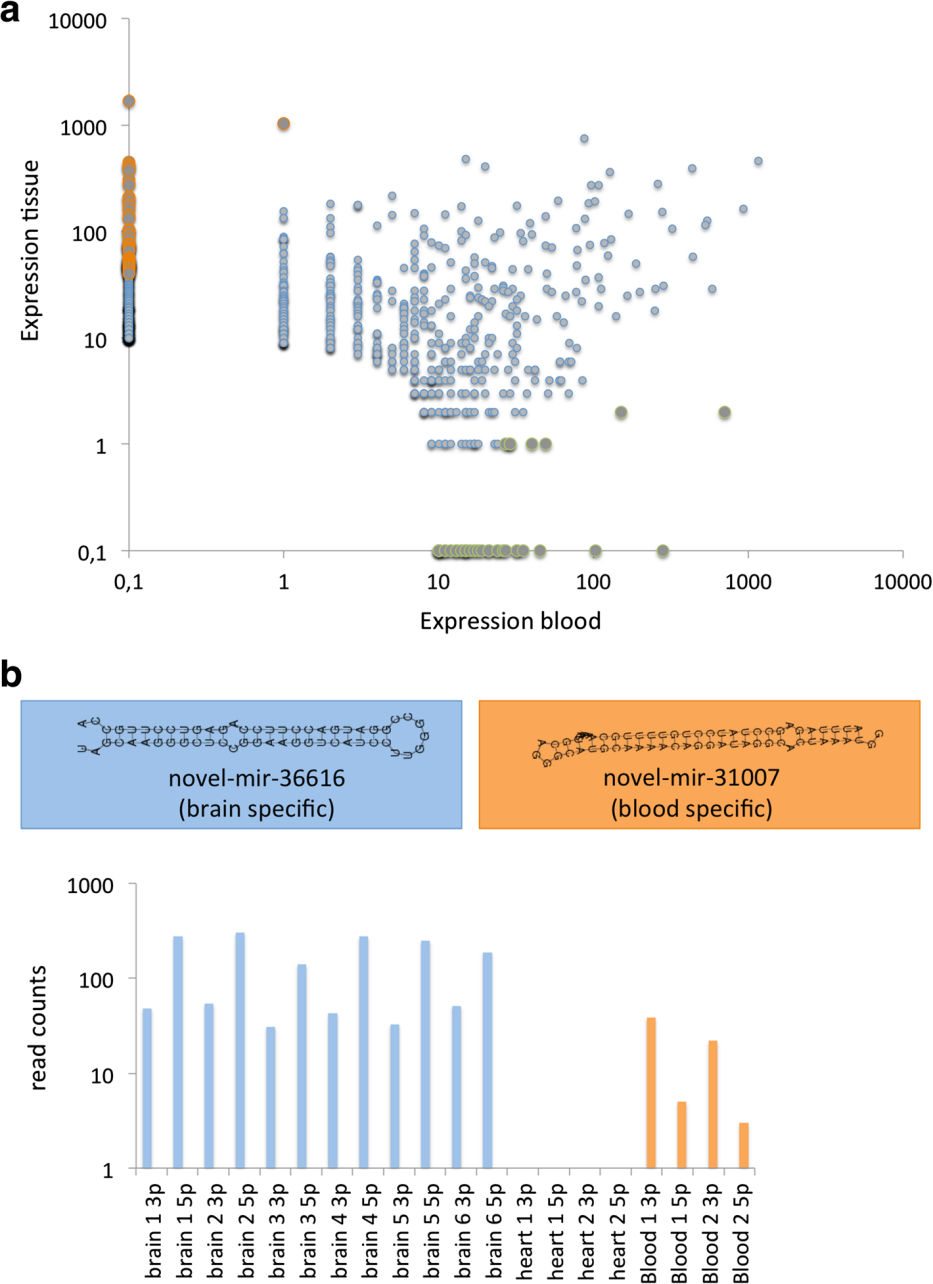
**a** Expression of novel miRNAs in blood versus tissue. The *green* miRNAs are specific for blood, the *orange* miRNAs for tissue, and the *blue* miRNAs were detected in both specimens. **b** Bar plot for two different miRNAs. The first miRNA novel-mir-36616 in *blue* is detected only in the brain tissue and not in the heart tissue or blood, the -3p form is one order of magnitude less expressed compared to the -5p mature form. The second miRNA novel-mir-31007 is expressed in blood and not in tissue, here, the -3p form is more than an order of magnitude more abundant compared to the -5p form

Predicting new miRNAs from NGS data is a challenging task since many false positive miRNA candidates are observed. We implemented our own prediction tool for miRNAs from NGS data and filtered the candidates stringently to reduce the false discovery rate. Without any filtering steps, our initial predictor trimmed for maximizing the ROC AUC returned 25,086 candidates across all samples. The exclusion of the candidates with low abundance (less than 10 total reads) reduced the number of candidates to around 10% (2354 candidates). Further analysis with *novoMiRank* (cutoff 1.5) filtered out more miRNAs, leaving 1553. The miRNAs were flagged by *novoMiRank* because of a high deviation from miRNAs in the first *miRBase* versions, including deviating length, free energy, or nucleic acid composition of miRNAs. Matching the remaining candidates to other RNA resource in a blacklisting step finally presented 926 miRNA candidates (Additional file 4: Table S2). Still, it is likely that this set contains many false positives. Additionally, low-throughput experimental validation of almost 1000 miRNA candidates, e.g., by Northern Blot is a very labor-extensive approach. We thus additionally compared the frequency of reads mapping to the blood versus tissue samples. As detailed in Fig. 5a, we observe a substantial variability between blood and tissue for the 926 miRNA candidates (correlation 0.18). Defining a miRNA as tissue/blood specific if it occurs with a factor of 100-fold higher in one of both sample types (normalized for the total number of samples) highlighted 74 new miRNA candidates specific for tissue and 36 new miRNA candidates specific for blood samples. Figure 5b shows bar plots for two miRNA precursors, the most tissue specific novel-mir-36616 (blue), only present in the brain samples, and the blood specific novel-mir-31007. The first miRNA, which is observed exclusively in the brain samples and not in the heart, reveals a significantly less expressed 3′ mature form as compared to the 5′ mature form. The second miRNA is exclusively observed in blood samples. Here, the 5′ mature form is lower expressed compared to the 3′ form. The boxes above the bar plots show the secondary structures of both miRNA candidates.

### miRNA target analysis

For all 926 miRNAs, we predicted targets using TargetScan. To rank miRNA-target interactions, we used the context++ score (distribution of the context++ score across all predictions is provided in Additional file 5: Figure S2). Thereby, we observed an accumulation of high-likelihood targets for tissue-specific miRNAs. Of the 926 miRNAs, the tissue specific had an average 42.8 targets, the neither for blood nor for tissue-specific miRNAs 40.7 targets while for blood-specific miRNAs, only 34.5 targets were predicted. The complex miRNA-target network is presented in Additional file 6: Figure S3. It contains 6014 nodes (5088 genes and 926 miRNAs). Network characteristics such as degree distribution and shortest path length are presented in Additional file 7: Figure S4. The genes with largest numbers of predicted miRNAs targeting the gene were CYB561D1 (229 miRNAs), FBXL12 (174 miRNAs), PML (162 miRNAs), and VNN3 (154 miRNAs). The distribution of miRNAs in the different group is presented as Venn diagram in Additional file 8: Figure S5). Among the predicted target genes that were found only for candidate miRNAs being blood specific was, e.g., HMOX1, heme oxygenase 1, mediating the first step of the heme catabolism by cleaving heme to build biliverdin or HPX, coding for hemopexin. The complex nature of the in silico calculated miRNA-target network requires further analyses to understand whether target genes accumulate in specific biochemical categories such as KEGG pathways or gene ontologies. We thus applied GeneTrail2 separately to the set of genes targeted by blood specific miRNAs, targeted by tissue specific miRNAs and by all other miRNAs. As the background sets, all genes predicted to be targeted by at least a single miRNA were selected and the functionality to compare different enrichment analyses by GeneTrail2 has been used. Enriched pathways seem to be largely relevant for either blood or tissue miRNAs, as Additional file 9: Figure S6 highlights. Tissue specific miRNAs had target genes enriched for DNA damage response, the apoptosis, or RNA polymerase II regulatory region DNA binding while blood miRNAs target genes were, e.g., enriched for TP35 network. Interestingly, tissue miRNA target genes also clustered on specific genomic locations (e.g., 19p12 and 19.q13) while blood miRNA targets did not show such an enrichment. In contrast, blood miRNA targets were enriched for disease phenotypes such as carotid artery diseases. In sum, the enrichment analysis highlights very distinct patterns for blood and tissue miRNA targets. Of course, not only the new miRNAs themselves but also the predicted targets deserve detailed experimental validation.

## Discussion

The advent of next-generation sequencing reduced the costs of sequencing while simultaneously increasing the speed of throughput [28]. Today, the costs for small RNA seq are almost equal to and even lower than miRNA microarrays, although small RNA-seq provides the additional possibility for detecting novel small RNA entities.

In the present study, we investigated two current sequencing approaches supporting massively parallel sequencing, which is of high relevance in small RNA research because of the high dynamic range of these molecules: DNA nanoball [11]-based sequencing by BGISEQ-500 and PCR cluster [8]-based sequencing by HiSeq. An important difference between these techniques is in that the first approach uses linear DNA amplification, and the second uses exponential DNA amplification to make sequencing arrays. The latter approach may in turn lead to amplification errors and some specific biases. Besides this fundamental difference, both approaches have their additional advantages and disadvantages. Specifically for the BGISEQ-500, the library preparation currently takes around three working days, the sequencing itself needs one or at maximum two working days. Each flowcell of the BGISEQ-500 has two lanes. On each of these lanes, 32 Gb data can be generated using single-end reads of length 50 bases. The cost of the reagent and material is around 200 USD for 20 million reads ensuring high-quality data at a reasonable cost.

Recently, we published a manuscript about bias in NGS and microarray analysis for miRNAs [6], highlighting that the expression of miRNAs on different platforms varies by, for example, the nucleic acid composition. In the validation by RT-qPCR, we focused on miRNAs discordant between the high-throughput platforms. Thereby, we observed cases where the RT-qPCR results were concordant with Illumina HiSeq, with microarrays or with none of the techniques. Therefore, we were especially interested how the BGISEQ-500 platform compares to the HiSeq platform and microarrays with the content from the *miRBase* for small RNA analysis.

Three miRNAs had high divergence between arrays and BGISEQ-500, among them hsa-miR-4454, which was high abundant in arrays but almost not detectable in BGISEQ-500. According to the miRBase, only 28% of users believe that this miRNA is real. Although such votes have only limited value, they at least indicate that this miRNA may be influenced by technological bias.

For high-throughput sequencing, the library preparation and the kits used play a crucial role for the quality of the sequencing results. Others and we noticed an overly abundance of the miRNA miR-486-5p when using the TruSeq kit (Illumina, San Diego), which seems to be independent of the source of the analyzed material [6, 29, 30]. Using the BGISEQ-500 platform, we observed lower read counts for this miRNA. However, in some cases, the miRNA abundance of BGISEQ-500 matches to the HiSeq sequencing results while microarrays show a different expression level, and in other cases, the BGISEQ-500 deviates from the other platforms and in several cases, all three techniques provide substantially divergent results. The more even distribution of reads of the BGISEQ-500 compared to the HiSeq results facilitates the discovery of new miRNAs, which are expected to be significantly less expressed as compared to the already known miRNAs, especially from early miRBase versions.

With respect to many miRNA currently annotated in miRBase and the rapidly growing number of new miRNAs, it is essential not only to have tools for filtering likely false-positives such as the NovoMiRank tool but also to carry out validation of miRNAs using other molecular biology approaches such as cloning and Northern blotting.

Focusing on the performance of the BGISEQ-500, we found a high technical reproducibility of sequencing results, which was however slightly below the technical reproducibility of microarrays. This fact can have different reasons, e.g., the different limit of detection of microarrays. In contrast to sequencing, microarrays have a saturation effect. With respect to the total number of discovered known miRNAs, performance of the BGISEQ-500 was comparable both to the Illumina and the microarray platform.

## Conclusions

In sum, none of the mentioned platforms seems to provide the “ultimate solution” in miRNA analysis. All have their advantages and disadvantages and show some bias for the detection of certain sequence types.

## Additional files

Additional file 1: Table S3. miRNA read count of the BGISEQ-500. (XLSX 250 kb)
Additional file 2: Figure S1. Predicted secondary structures for selected miRNAs. (PNG 241 kb)
Additional file 3: Table S1. Comparison of BGISEQ-500 to Agilent. (XLSX 135 kb)
Additional file 4: Table S2. List of novel miRNA candidates. (XLSX 6531 kb)
Additional file 5: Figure S2. Histogram of the decade logarithm of the context++ scores (multiplied by −1) of predicted targets for the candidate miRNAs. Since negative context++ scores are favorable, the miRNA targets on the right of the diagram are more likely true interactions. (PNG 78 kb)
Additional file 6: Figure S3. Full interaction network. Predicted miRNAs are represented in large nodes, colored by type (red: blood specific, blue: tissue specific, green: all others) and genes are represented by smaller gray nodes. (PNG 1033 kb)
Additional file 7: Figure S4. Core network characteristics as node degree distribution (*top*) and shortest path length (*bottom*). (PNG 129 kb)
Additional file 8: Figure S5. Venn diagram showing the distribution of predicted target genes for tissue-specific miRNA candidates, blood-specific miRNA candidates, and all other miRNA candidates. (PNG 156 kb)
Additional file 9: Figure S6. Comparison of the pathway enrichment analysis for the GeneTrail2 analysis with respect to the three target sets. *Red arrows* represent significant enrichments. (PNG 289 kb)
