# Supplementary figures and images for: cPAS-based sequencing on the BGISEQ-500 to explore small non-coding RNAs
Source: Clin Epigenetics. 2016 Nov 21;8:123. doi: 10.1186/s13148-016-0287-1 (PMC5117531; doi:10.1186/s13148-016-0287-1)

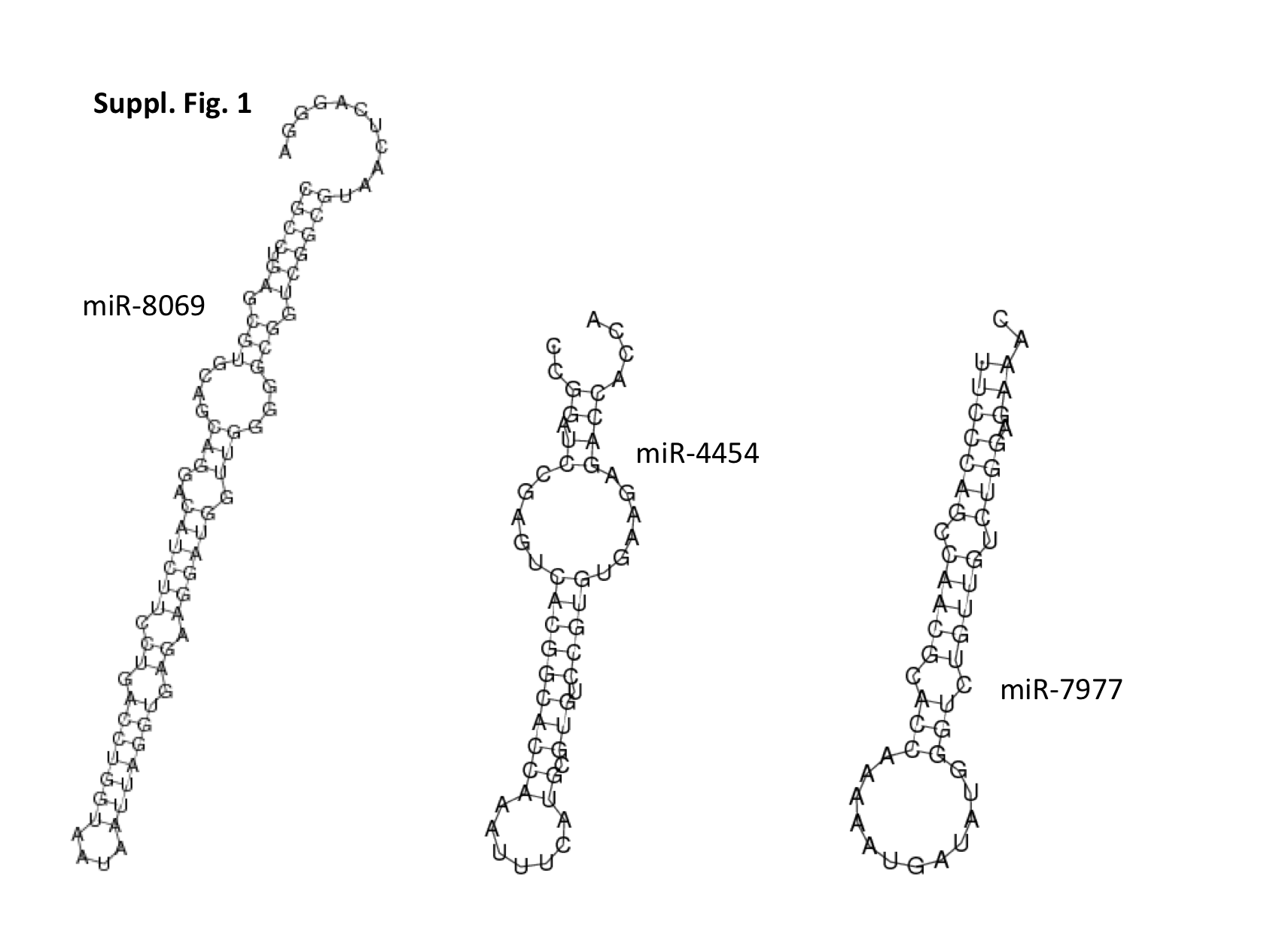

Supplement: Additional file 2: Figure S1. — Predicted secondary structures for selected miRNAs. (PNG 241 kb) [file 13148_2016_287_MOESM2_ESM.png]

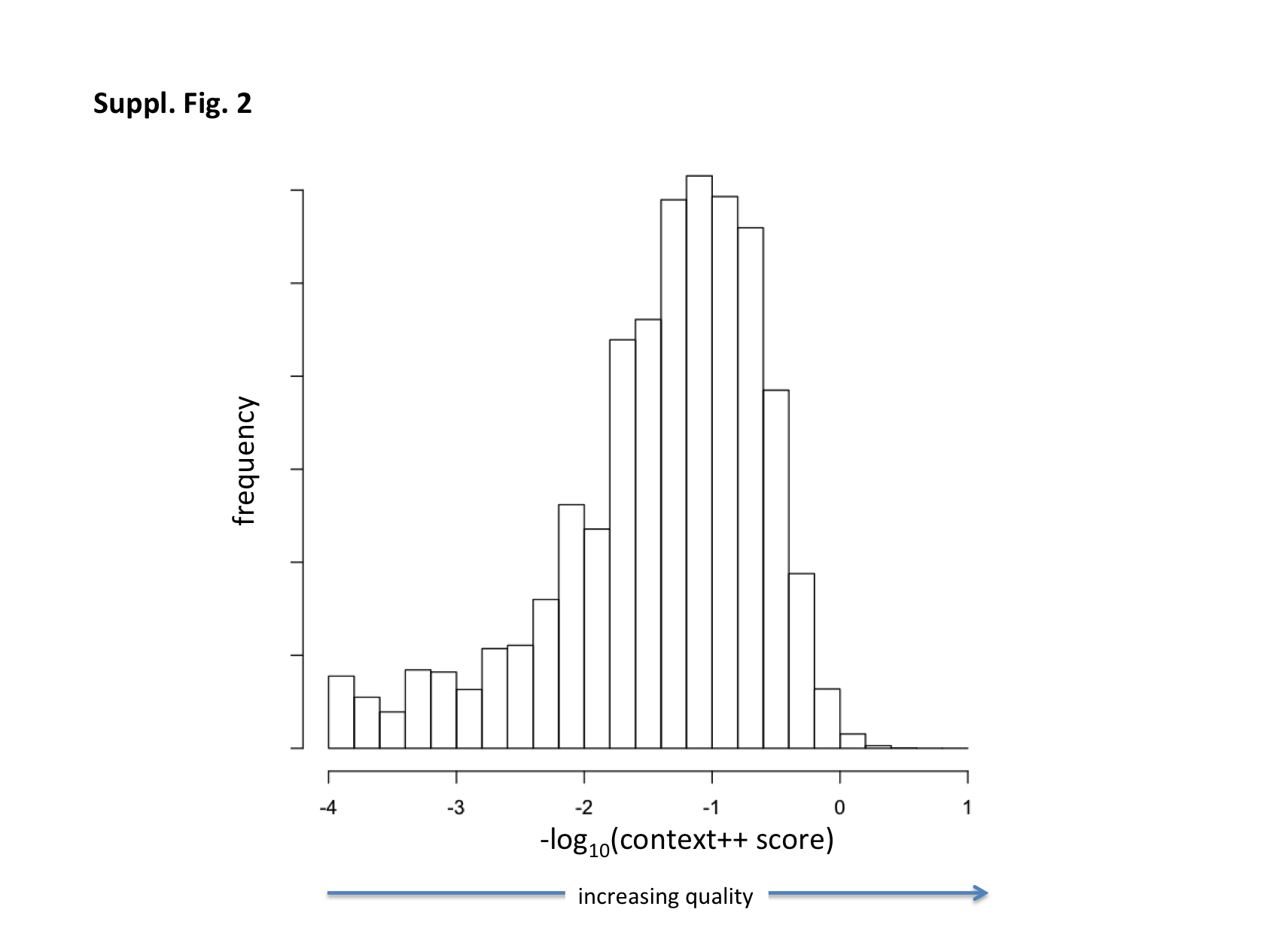

Supplement: Additional file 5: Figure S2. — Histogram of the decade logarithm of the context++ scores (multiplied by −1) of predicted targets for the candidate miRNAs. Since negative context++ scores are favorable, the miRNA targets on the right of the diagram are more likely true interactions. (PNG 78 kb) [file 13148_2016_287_MOESM5_ESM.png]

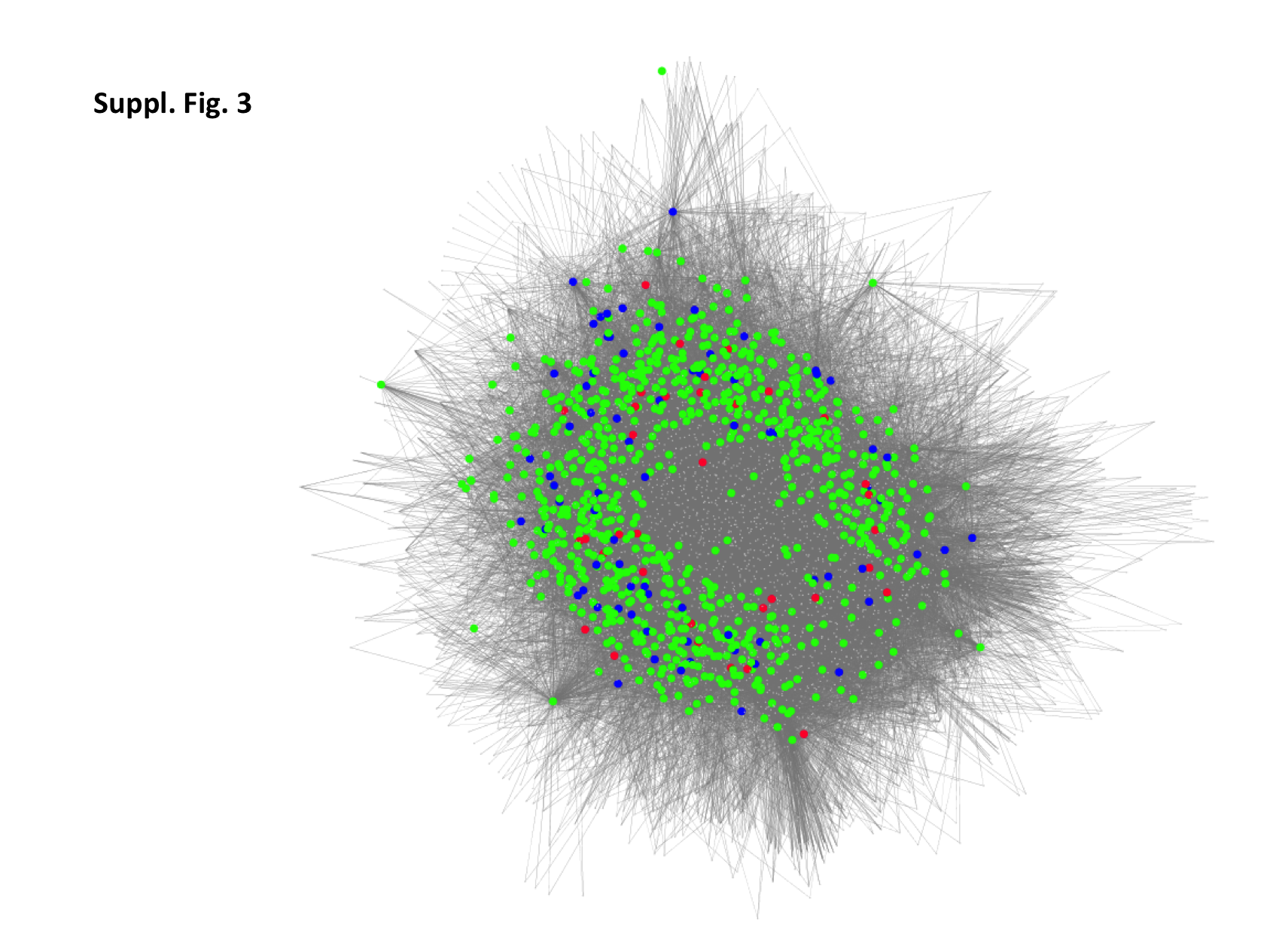

Supplement: Additional file 6: Figure S3. — Full interaction network. Predicted miRNAs are represented in large nodes, colored by type (red: blood specific, blue: tissue specific, green: all others) and genes are represented by smaller gray nodes. (PNG 1033 kb) [file 13148_2016_287_MOESM6_ESM.png]

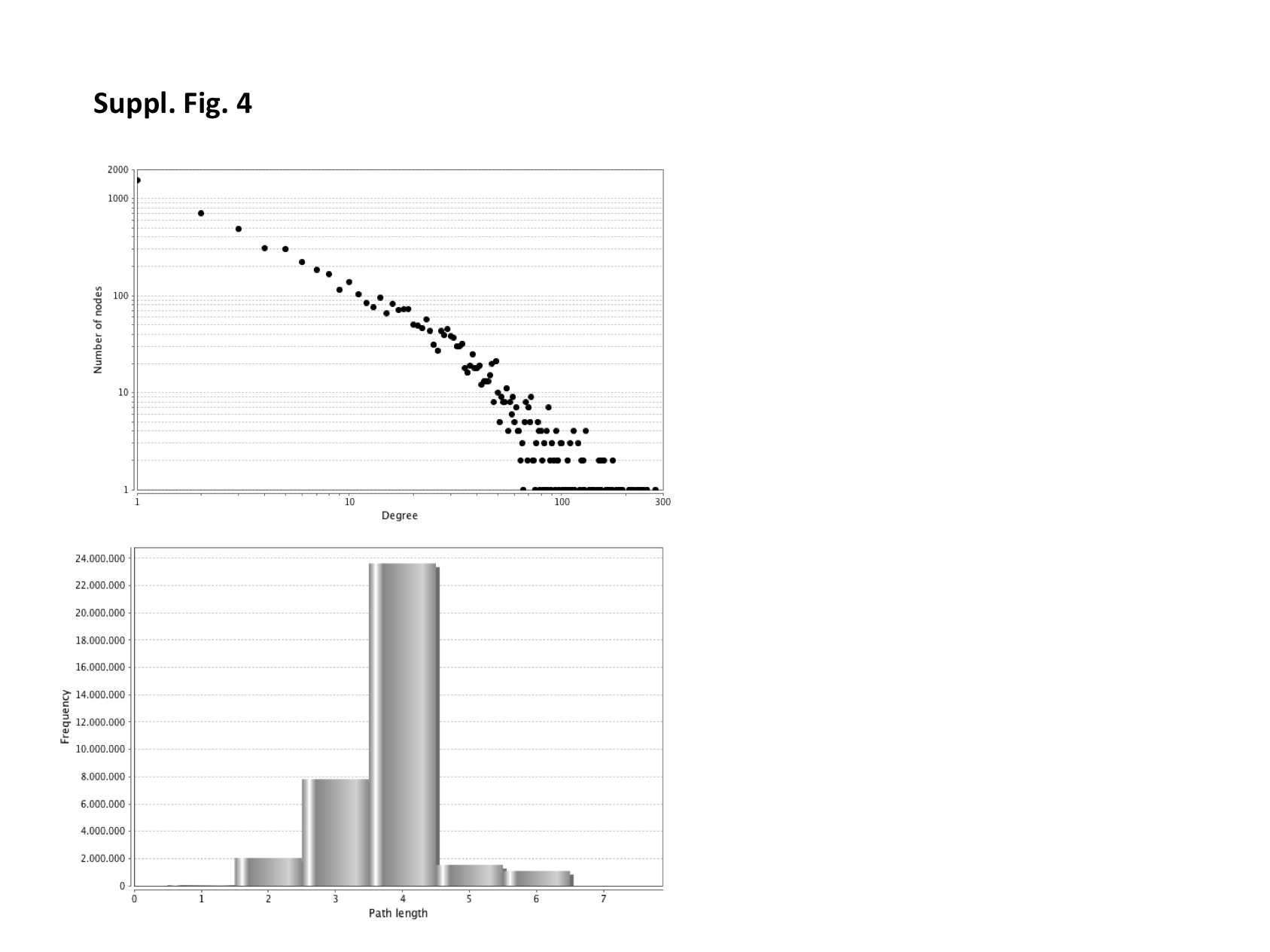

Supplement: Additional file 7: Figure S4. — Core network characteristics as node degree distribution (top) and shortest path length (bottom). (PNG 129 kb) [file 13148_2016_287_MOESM7_ESM.png]

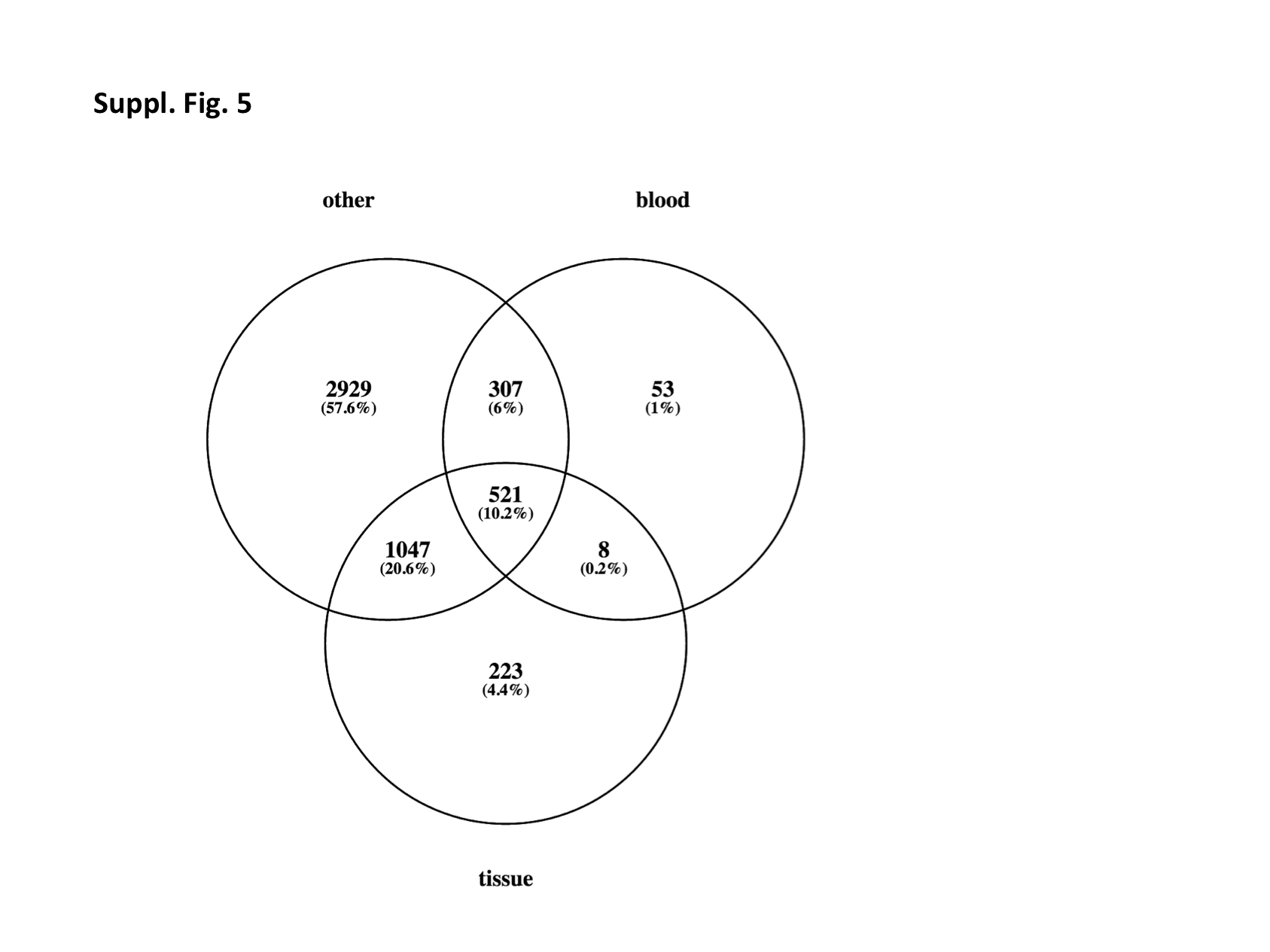

Supplement: Additional file 8: Figure S5. — Venn diagram showing the distribution of predicted target genes for tissue-specific miRNA candidates, blood-specific miRNA candidates, and all other miRNA candidates. (PNG 156 kb) [file 13148_2016_287_MOESM8_ESM.png]

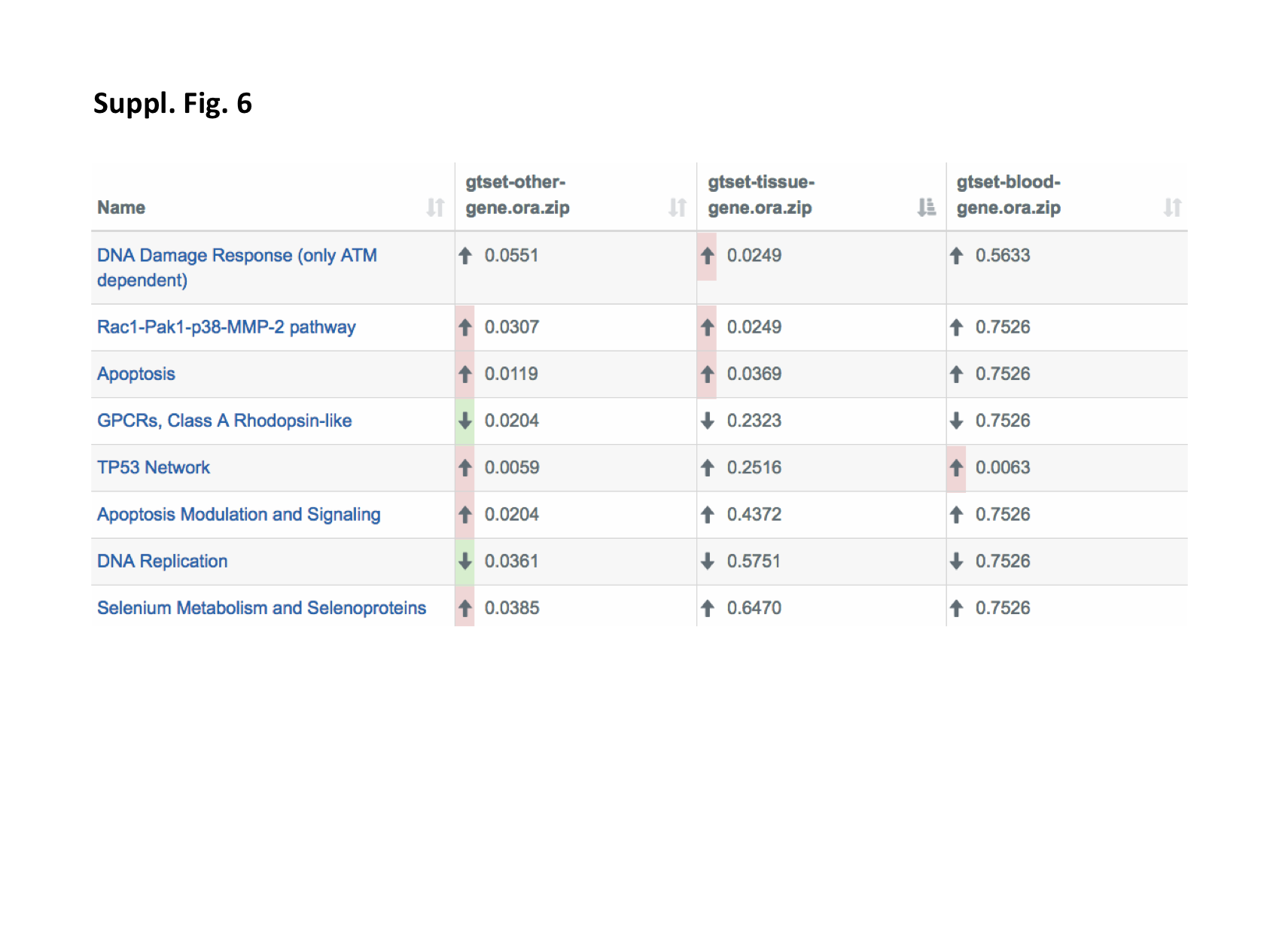

Supplement: Additional file 9: Figure S6. — Comparison of the pathway enrichment analysis for the GeneTrail2 analysis with respect to the three target sets. Red arrows represent significant enrichments. (PNG 289 kb) [file 13148_2016_287_MOESM9_ESM.png]
